# Retraction for Huang et al., “Long-Read Metagenomics of Marine Microbes Reveals Diversely Expressed Secondary Metabolites

**DOI:** 10.1128/spectrum.02381-24

**Published:** 2026-01-29

**Authors:** Ranran Huang, Yafei Wang, Daixi Liu, Shaoyu Wang, Haibo Lv, Zhen Yan

## RETRACTION

Volume 11, no. 4, e01501-23, 2023, https://doi.org/10.1128/spectrum.01501-23. We sincerely regret to announce the retraction of our article “Long-Read Metagenomics of Marine Microbes Reveals Diversely Expressed Secondary Metabolites”. This decision, though difficult, is deemed necessary in light of significant concerns regarding the integrity of the work.

Upon thorough review, we recognize that the article contains similarities in both structure and content to previously published works: *Biosynthetic potential in Antarctic soil bacteria revealed through long-read metagenomic sequencing* (1), *Long-read metagenomics of soil communities reveals phylum-specific secondary metabolite dynamics* (2), and *Diverse secondary metabolites are expressed in particle-associated and free-living microorganisms of the permanently anoxic Cariaco Basin* (3). This overlap arose primarily from our negligence during the preparation of the manuscript. While our data incorporated metatranscriptomic analyses, we inadvertently drew upon certain elements of the referenced study, constituting a serious oversight.

We extend our sincere apologies to the authors of the referenced work, our peers, and the broader scientific community for this oversight. We commit to ensuring that such errors do not occur in future publications and will strive to improve our research practices.
